# Hydrocephalus and Intracranial Hypertension by an Odontogenic Brain Abscess

**DOI:** 10.7759/cureus.26945

**Published:** 2022-07-17

**Authors:** Rogério P Amorim, Vinícius M Henriques, Francisco T Junior, Vinícius G Reis, Stephanie O Bulhões

**Affiliations:** 1 Neurological Surgery, Gaffrée and Guinle University Hospital (HUGG), Rio de Janeiro, BRA; 2 Neurosurgery, Gaffrée and Guinle University Hospital (HUGG), Rio de Janeiro, BRA; 3 Neurological Surgery, Hospital de Força Aérea do Galeão (HFAG), Rio de Janeiro, BRA

**Keywords:** intracranial hypo-hypertension, adult hydrocephalus, tooth extraction, streptococcus intermedius bacteremia, brain abscess

## Abstract

Brain abscesses are rare and severe infections with high mortality, corresponding to neurosurgical emergencies. 12% of all abscesses are caused by odontogenic etiologies, either an infectious process or a dental procedure. This paper describes a case of a patient who underwent tooth extraction, presenting days later with hydrocephalus and intracranial hypertension due to a brain abscess, whose isolated pathogen is the same identified in the oral cavity.

## Introduction

Brain abscesses are severe and potentially fatal infections that correspond to neurosurgical emergencies. Its incidence is estimated to be one to eight cases per 100,000 patients in the United States [1], and its etiology is varied and may arise from bacterial dissemination from a distant primary focus or from a direct contiguous invasion of an adjacent site. In most patients, the lesion is associated with predisposing factors, such as human immunodeficiency virus infection; use of immunosuppressive medications; history of trauma, dental infection, or surgical procedure; or disseminated systemic infection such as endocarditis or sepsis [1-3].

Odontogenic infections encompass, in addition to gingivitis and periodontitis, dental procedures such as tooth extractions, endodontic treatments, and oral surgery. They are usually confined within the dental socket or periodontium, but rarely spread leading to more severe infections such as cavernous sinus thrombosis, brain abscess, and endocarditis [2-5].

Less than half of the patients with odontogenic brain abscesses have intraoral symptoms or a history of dental procedures prior to the neurological clinic. Of these patients, the main symptoms are: headaches; disorientation; motor deficit; convulsive crisis; and reduced level of consciousness. The mean time between the onset of neurological symptoms after dental procedures is 17.6 days [2,6].

The diagnosis is based on three principles: the identification of a brain abscess with a pathogen found in the oral cavity; the absence of other possible sources of dissemination; and history of oral infections and/or dental procedures. Due to the rarity of odontogenic origin, an oromaxillofacial investigation should always be conducted to exclude the diagnosis, as less than half of the blood cultures and cultures of abscess contents are positive. The management in each case comprises the treatment already established for brain abscesses in general, involving antibiotic therapy and surgical drainage in early selected cases [2,5,6]. This study aims to describe a case of odontogenic brain abscess with atypical clinical and neurosurgical complications, with a favorable outcome and resolution of the clinical condition.

## Case presentation

**Figure 1:**
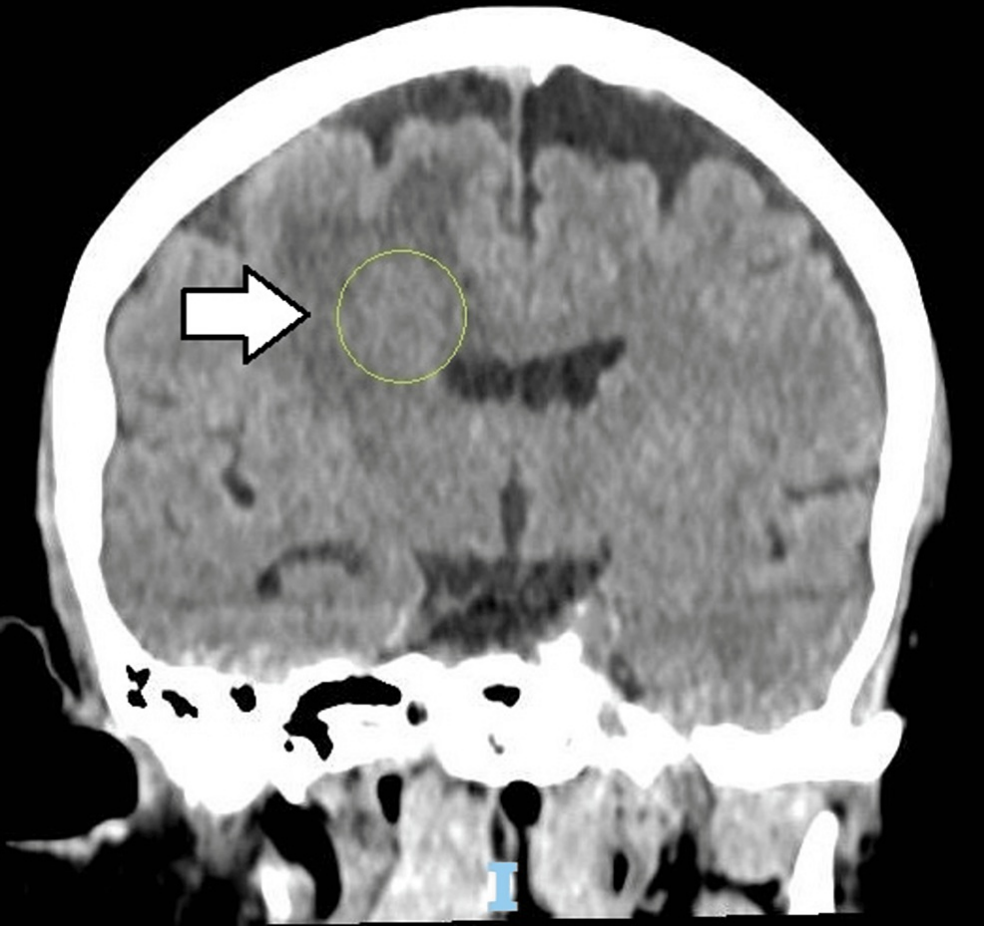
Brain CT scan showing a 1.4x1.3x1.9 cm heterogeneous mass (green circle) at the right frontal lobe (arrow)

**Figure 2:**
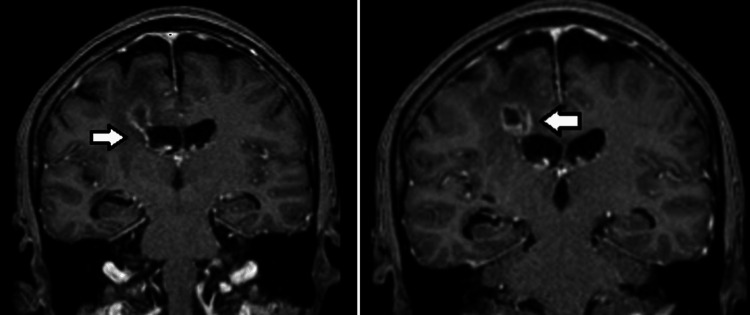
Brain T1-weighted MRI showing a peripheral contrast uptake e signs of diffusion restriction, suggesting purulent content associated to perilesional edema (arrow on left and right images)

**Figure 3:**
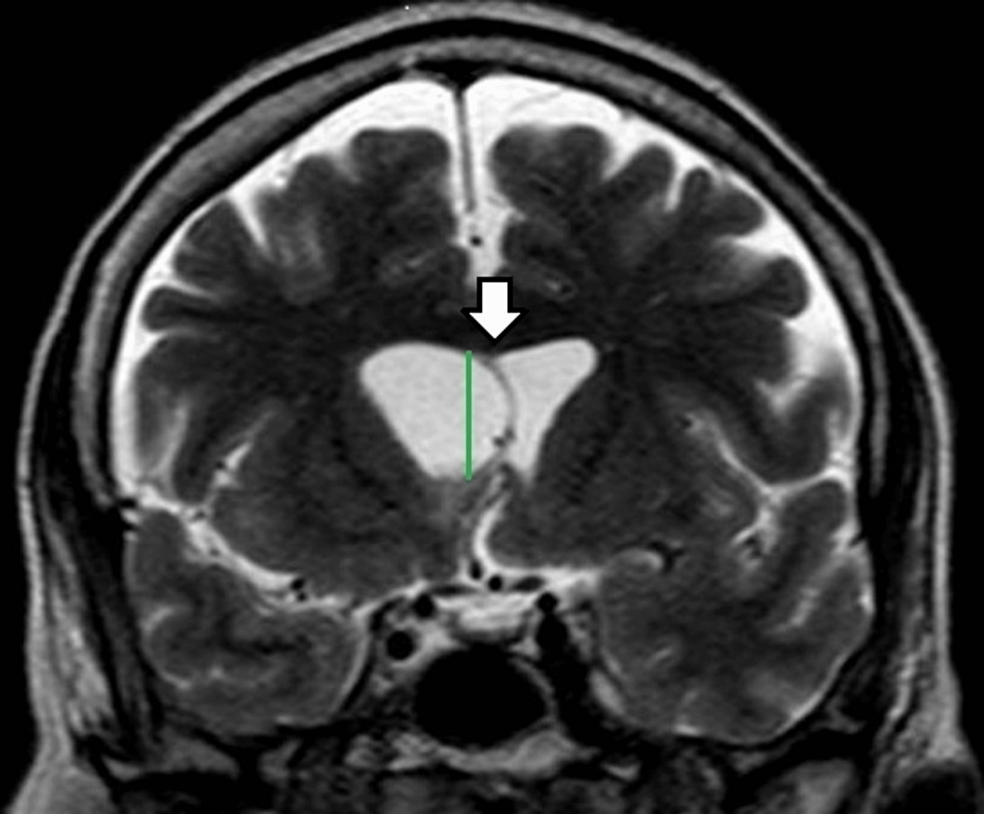
Brain T2-weighted MRI showing dilatation of the ventricular system and deviation of the septum pellucidum to the left (arrow showing the septum pellucidum and green line showing midline)

A 67-year-old female, without comorbidities, is admitted to the emergency unit with severe headache and neck pain, without fever, in the previous 24 hours, with a history of long-term migraine and tooth extraction of the upper left second molar 10 days before, without antibiotic prophylaxis. On neurological examination, she had a Glasgow coma score equal to 15, with Kernig and Brudzinski signs and no signs of infection in the oral cavity. Lumbar puncture was performed showing turbid cerebrospinal fluid (CSF) with opening pressure equal to 25 mmHg, 35 mg/dL of glycemia, hyperproteinorrachia, and 170 cells/mm³ with polymorphonuclear predominance. After the lumbar puncture, treatment with ceftriaxone was initiated. A computed tomography (CT) scan of the skull was performed, which showed a lesion with ill-defined limits and heterogeneous density, located in the right frontal lobe measuring 1.4x1.3x1.9 cm, with a slight mass effect on the body of the right lateral ventricle (Figure 1). There was complementation with Magnetic Resonance Imaging (MRI), which observed on the T1-weighted, a ring uptake of contrast and signs of diffusion restriction, suggesting purulent content, which infiltrated the right lateral ventricle wall (Figure 2), promoting dilatation of the ventricular system and deviation of the septum pellucidum to the left (Figure 3).

After two days, the patient started to projectile vomit, worsening headache, and disorientation, with a drop in the Glasgow coma score to 13 points, being taken to the intensive care unit for surveillance and submitted to a new CT scan of the head, demonstrating an increase in the nodular lesion with extension to the ependymal region and the frontal horn of the ipsilateral ventricle, associated with greater perilesional edema and greater ventricular dilation. The patient then underwent placement of an external ventricular drain and an intracranial pressure (ICP) monitoring catheter. During the procedure, the ICP was equal to 16 mmHg and the presence of CSF contaminated with the purulent collection was observed, which was collected for analysis. The patient was then admitted to the intensive care unit, on mechanical ventilation, without tolerating weaning from ventilation. CSF culture and the oropharyngeal swab identified a ceftriaxone-sensitive Streptococcus intermedius.

**Figure 4:**
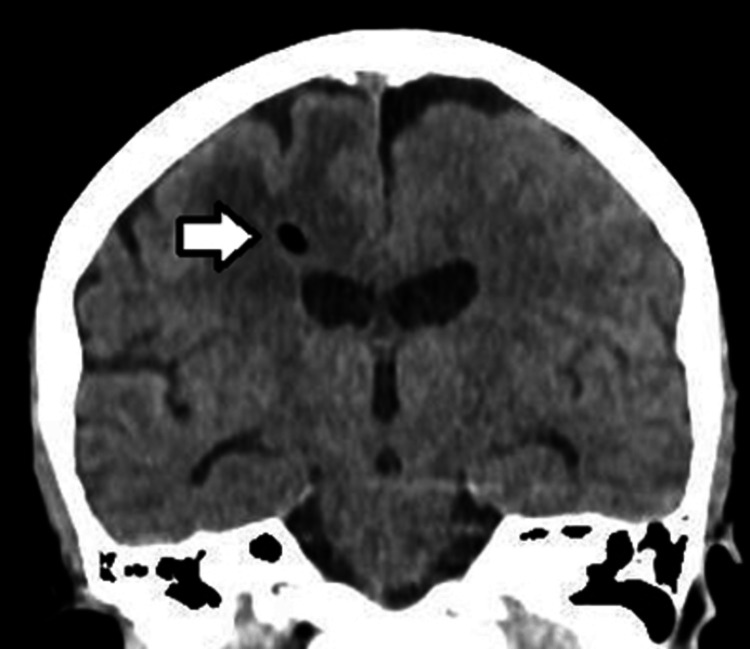
Brain CT scan showing resolution of the brain abscess and the hydrocephalus, with a small area of residual pneumoenchepalon (arrow)

The patient showed progressive clinical improvement, with the CSF becoming clear, the ICP evolving to 6 mmHg, and improving the inflammatory parameters. The ICP monitoring catheter was removed in three days and the patient was extubated four days after the surgical procedure and transferred to the infirmary, two days later. The stay in the ward lasted an additional 15 days for neurological surveillance and antibiotic control, with the patient being discharged from the hospital without deficits or complaints, with complete resolution of the infectious lesion and a new brain CT scan showing resolution of the brain abscess and the hydrocephalus, with a small area of residual pneumoenchepalon (Figure 4). The patient's reassessment was performed on three occasions, two weeks, three months, and six months after hospital discharge, with the complete improvement of symptoms, without new clinical manifestations.

## Discussion

Brain abscesses are rare, severe, and potentially fatal infections composed of restricted areas of suppuration with cerebral involvement, with mortality between 5% and 32% [7]. Most cases involve men under 60 years of age and are rarely seen in children, which differs from our case which describes a 67-year-old woman [2,3,8-10].

Among the possible causes, odontogenic is one of the rarest, corresponding to approximately 12% of all brain abscesses [2,8-11]. Most intracranial lesions associated with infections of oral origin involve cavernous sinus thrombosis, sometimes associated with brain abscesses. Isolated abscesses are much less frequent, while subdural empyemas are much rarer [2,3,8-11].

The first description of the odontogenic abscess was made in 1925 by Bolton et al. and the first presentation of a series of cases in 1945 by Haymaker et al. [9,12]. However, until now, there are less than 150 single cases published on the subject, which is explained by the fact that less than half of the patients had intraoral symptoms or a history of dental procedures prior to the neurological clinic. Some factors would be associated with a higher risk of intracranial suppuration, such as those with a history of maxillary molar extraction, immunosuppression, or cyanotic heart disease [2,6,9,12,13].

The oral cavity harbors an extensive and diverse microflora, with more than 500 types of bacteria described that can initiate an infectious process. Odontogenic infections encompass not only conditions such as gingivitis and periodontitis, but also dental procedures such as tooth extractions, endodontic treatments, and oral surgery. They are usually confined within the dental socket or periodontium, however, they can spread leading to more severe infections such as cavernous sinus thrombosis, brain abscess, and endocarditis. They rarely lead to intracranial infections, with hematogenous dissemination being the main pathophysiological route of infection [2-5].

The possibility that these procedures induce bacteremia depends on several factors, such as the amount and complexity of the resident microflora, the severity of inflammation in the adjacent tissues, and the immunological impairment of the patient. Some patients already have bacteremia before dental treatment, with a higher prevalence after the procedure and depending on oral hygiene. The entry of these microorganisms into the skull can be by contiguity, hematogenous dissemination, local lymphatic system, and indirectly by extraoral odontogenic infection [3,4,6,10].

Some bacteria such as Actinomyces sp, Fusobacterium, and Streptococcus are saprophytes of the oral cavity and can generate different types of periodontal diseases under favorable conditions. When in other topographies, opportunistic infections can happen, being identified by the culture of the material collected. During the etiological investigation, approximately 30% of abscesses are polymicrobial and the main bacteria isolated belong to the streptococci group, and in 20% of cases, it is not possible to identify a pathogen. Other more uncommon pathogens have also been reported, such as Aggregatibacter aphrophilus [14], Filifactor alocis, Porphyromonas gingivalis [15], Parvimonas micra [16], and some types of fungus [10]. In 17% of the cases, it is possible to isolate the same agent from the orodental and cerebral sites, and in the literature, only 11.35% of the studies investigated both foci. CSF culture and an oropharyngeal swab from our patient identified Streptococcus intermedius, a pathogen that has been described as associated with other suppurative processes such as diverticular abscess [17], and which belongs to the most frequent microbial group in odontogenic abscesses [12].

Among patients who develop clinical manifestations, the main complaint is headache, which may be associated with disorientation, motor deficit, convulsion, and reduced level of consciousness. The mean time between the onset of neurological symptoms after dental procedures is 17.6 days. The severity of the symptoms is explained by the location of the lesion and the inflammatory effects of the suppurative process, with the worst prognosis, progressing to coma and death, mainly associated with the infection itself. To the best of our knowledge, this is the first case that describes an obstruction of CSF flow leading to hydrocephalus and intracranial hypertension in the setting of an odontogenic brain abscess [2,6].

The management of these infections requires multidisciplinary attention, involving an infectious disease specialist, neurologist, neuroradiologist, neurosurgeon, and dentist. The aim is to eliminate the infectious process and reduce the mass effect caused by the necrotic tissue, the suppurative inflammatory response, and the surrounding brain edema. The first description of conservative management of brain abscesses was given by Heineman et al. in 1971 [18], using antibiotics without a surgical approach, opening the way for further studies that demonstrate the success in the non-surgical treatment of brain abscesses, using antibiotic therapy alone. Among the conservative indications, we include small abscesses (<2 cm in diameter) and abscesses in the cerebritis stage, as is the case of our patient who had a small lesion and did not have her lesion drained, resolved only with the use of ceftriaxone. The surgical procedure in our case aimed to treat intracranial hypertension and not the abscess, guaranteeing time for the patient to respond to antibiotic therapy [1,10,19,20].

The prevention of this type of abscess is based on routine dental care and the habit of good oral hygiene. In all patients, antibiotic prophylaxis is recommended before any dental procedure, usually with penicillin or cephalosporin, and especially in those with risk factors. It is evident in our case that the absence of prophylaxis was one of the factors that culminated in the infectious spread [3,8,10].

## Conclusions

Brain abscesses are rare and potentially fatal infections composed of restricted areas of suppuration with cerebral involvement, with mortality between 5% and 32%. The odontogenic etiology composes 4% to 12% of all cases of this infection and is related to dental procedures and orodental infections. Good control and dental care are essential to reduce the risks and incidence of oral pathology. One of these essential care is antibiotic prophylaxis during dental procedures, reducing the morbidity and mortality of patients who develop and complicate bacteremia and distant infections.
